# 4144 Identifying the needs of family caregivers of people with dementia to improve service delivery: Bridging a research-practice gap

**DOI:** 10.1017/cts.2020.401

**Published:** 2020-07-29

**Authors:** Sara A. Moss, Lauren E. Gebhardt-Kram, Holly Dabelko-Schoeny, Jennifer Cheavens

**Affiliations:** 1 The Ohio State University

## Abstract

OBJECTIVES/GOALS: The goal of this mixed methods project is to develop a comprehensive framework of the personal and care-related needs of informal caregivers of people with dementia. This model can be used to enhance targeted delivery of evidence-based services to caregivers in need. METHODS/STUDY POPULATION: To create a model of the personal and care-related needs of family caregivers of people with dementia, we conducted semi-structured in-depth interviews with current caregivers (*N* = 12) and conducted a content analysis of materials related to government reports and evidence-based interventions (*N* = 28) and existing measures of dementia caregiver needs (*N* = 54). Content analysis is a systematic qualitative methodology that is used to distill complex source material into content-related categories and is well-suited for both interview data and document data (Elo & Kyngäs, 2008). We identified themes related to caregiver needs through examination of materials within and across source categories. RESULTS/ANTICIPATED RESULTS: We propose a framework of five inter-related need categories: Environmental needs (e.g., transportation, health-care access, financial resources, time), psychological needs (e.g., emotional wellbeing, identity/autonomy, perceived preparedness), social needs (e.g., social support, family dynamics), health-related needs (e.g., health behaviors, sleep), and needs related to the care and functioning of the person with dementia. We also consider how needs and background characteristics transact to influence which services may be of greatest use. In the future, we plan to test this model empirically with a nationally representative sample of caregivers. DISCUSSION/SIGNIFICANCE OF IMPACT: Evidence-based services exist to meet the needs of dementia caregivers. A dearth of models clearly defining caregiver needs limits empirically-based plans for dissemination of services. We have identified a framework of the needs of family caregivers from which to create targeted dissemination plans.

